# Induction of Mast-Cell Accumulation by Promutoxin, an Arg-49 Phospholipase A_2_

**DOI:** 10.1155/2013/206061

**Published:** 2012-12-20

**Authors:** Ji-Fu Wei, Xiao-Long Wei, Ya-Zhen Mo, Haiwei Yang, Shaoheng He

**Affiliations:** ^1^Clinical Experiment Center, The First Affiliated Hospital of Nanjing Medical University, Nanjing 210029, China; ^2^Allergy and Inflammation Research Institute, The Shantou University Medical College, Shantou, Guangdong 515041, China; ^3^Research Division of Clinical Pharmacology, The First Affiliated Hospital of Nanjing Medical University, Nanjing 210029, China

## Abstract

Local inflammation is a prominent characteristic of snakebite wound, and snake-venom phospholipase A_2_s (PLA_2_s) are some of the main component that contribute to accumulation of inflammatory cells. However, the action of an R49 PLA_2_s, promutoxin from *Protobothrops mucrosquamatus* venom, on mast-cell accumulation has not been previously examined. Using a mouse peritoneal model, we found that promutoxin can induce approximately-6-fold increase in mast-cell accumulation, and the response lasts at least for 16 h. The promutoxin-induced mast cell accumulation was inhibited by cyproheptadine, terfenadine, and Ginkgolide B, indicating that histamine and platelet-activating factor (PAF) is likely to contribute to the mast-cells accumulation. Preinjection of antibodies against adhesion molecules ICAM-1, CD18, CD11a, and L-selectin showed that ICAM-1, and CD18, CD11a are key adhesion molecules of promutoxin-induced mast-cell accumulation. In conclusion, promutoxin can induce accumulation of mast cells, which may contribute to snake-venom wound.

## 1. Introduction

snake-venom phospholipase A_2_s (PLA_2_s) are low-molecular-weight (13,000–14,000 Da), secretory phospholipases containing seven disulfide bonds. Usually, the PLA_2_s from Crotalidae or Viperidae venom are divided into two major groups: the Asp-49 PLA_2_s (D49 PLA2s) and Lys-49 PLA_2_s (K49 PLA_2_s), and several minor groups: Ser-49 PLA_2_s (S49 PLA_2_s) [1–3], Asn-49 PLA_2_s (N49 PLA_2_s) [4, 5], and Arg-49 PLA_2_s (R49 PLA_2_s) [6–8]. Besides the digestive function, snake PLA_2_s exhibit several other pharmacological properties including antiplatelet [9, 10], anticoagulant [11], hemolytic [9], neurotoxic (presynaptic) [12], and myotoxic [13–15] properties. They have also been involved in various inflammatory processes such as edema, inflammatory cell infiltration, and mast-cell activation [15–20]. 

Mast cells are primarily located in mucosal and perivascular areas of various tissues, which play an important role in body-defense processes. Mast cells can be activated by snake-venom and release carboxypeptidase A and possibly other proteases, which can degrade venom components [21, 22]. Several snake-venom PLA_2_s were reported to be able to activate the rat mast cells and to induce microvascular leakage and inflammatory-cell accumulation at the sites of inflammation [15–20]. Our previous studies showed that TM-N49, an N49 PLA_2_ purified from *Protobothrops mucrosquamatus *venom, induces skin edema and mast-cell activation and accumulation [23], and promutoxin, an R49 PLA_2_ purified from the same snake, can activate mast cells and induce skin edema [24]. Both TM-N49 and promutoxin are devoid of catalytic activity and are thought to contribute to *Protobothrops mucrosquamatus* venom toxicity [5, 8]. Moreover, both TM-N49 and promutoxin are potent stimuli for induction of neutrophil, lymphocyte, macrophage, and eosinophil accumulation in the mouse peritoneum [25]. These observations suggested that the two novel subgroups of group II PLA_2_ may contribute to the inflammatory process caused by snake-venom poisoning. However, the ability of R49 PLA_2_ on induction of mast-cell accumulation has not yet been reported. In the present study, we investigated the mechanisms of promutoxin-induced mast-cell accumulation. 

## 2. Materials and Methods

### 2.1. Reagents

*Protobothrops mucrosquamatus* crude venom was obtained from the stock of the Kunming Institute of Zoology, the Chinese Academy of Sciences. SP-sephadex C-25, heparin sepharose (FF) and superdex 75 were obtained from LKB Pharmacia (Uppsala, Sweden). The following compounds were purchased from Sigma (St. Louis, USA): egg phosphatidyl choline, Triton X-100, trifluoroacetic acid, honey-bee venom phospholipase A_2_, platelet-activating factor (PAF), cyproheptadine, and ginkgolide B. Quinacrine was obtained from calbiochem (San Diego, CA, USA). Reagents for sodium dodecyl-sulphate-polyacrylamide gel electrophoresis (SDS-PAGE) were obtained from Bio-Rad Laboratories Inc. (Hercules, USA). Coomassie Plus assay kit was purchased from Pierce Chemical Co. (Rockford, IL, USA). Fetal-calf serum (FCS) and minimum essential medium (MEM) containing 25 mM *N*-2-hydroxyethylpiperazine-*N *′-2-ethane sulphonic acid (HEPES) were purchased from Gibco (Paisley, Renfrewshire, UK). Rat monoclonal antibodies, anti-mouse CD 11a lymphocyte function-associated antigen 1 (LFA-1) *α* chain, clone M17/4; anti-mouse CD 62L (L-selectin), clone MEL-14; anti-mouse CD18 (integrin *β*_2_ chain), clone M18/2; rat IgG2a isotype standard, clone R35-95; hamster anti-mouse CD54 intercellular adhesion molecule 1 (ICAM-1) monoclonal antibody, clone 3E2; hamster IgG1 isotype standard, clone A19-3 were obtained from BD Biosciences Pharmigen (CA, USA). Hepes and all other chemicals were of analytical grade. BALB/c mice (20–25 g) were bred and reared under strict ethical conditions according to international recommendation. 

### 2.2. Purification of Promutoxin

Promutoxin was isolated from *Protobothrops mucrosquamatus *crude venom following the procedures described previously [8]. Briefly, the lyophilized venom (1.5 g) was dissolved in 20 mL of 0.05 M sodium phosphate buffer (pH 5.8) before being loaded on an SP-Sephadex C-25 column. The absorbed proteins were eluted with a linear gradient (0–0.8 M NaCl). Peak 7 was collected and loaded on Superdex 75 column in 25 mM sodium phosphate buffer (pH 5.8 with 0.15 M NaCl). The main peak was collected, and then loaded on a reverse-phase C_18_ high-performance liquid chromatography (HPLC) (Waters Corporation, Milford, MA, USA). The main peak fraction, representing the purified promutoxin, was pooled, lyophilized, and stored at –20°C.

Protein concentration was determined by using a Coomassie Plus assay kit with BSA as standard. The PLA_2_ activity was routinely assayed by a titration method using egg yolk as substrate [26] and by a colorimetric assay using L-phosphatidylcholine as substrate [27]. Honey-bee PLA_2_ was employed as positive control.

### 2.3. Induction of Mast-Cell Accumulation

Various doses of promutoxin, BSA or normal saline were injected in 0.5 mL volumes into the peritoneum of male BALB/c mice, whose abdominal skin was swabbed with 70% ethanol, a group of 6 mice for each dose. This model was adapted from that described by Thomas and colleagues [28], which complied with the European Community guidelines for use of experimental animals. At 10 min, 2 h, 6 h, or 16 h following injection, animals were sacrificed by inhalation of carbon dioxide, and their peritoneal lavages were collected following a standardized procedure with 5 mL normal saline. After centrifugation at 500 g for 10 min at 4°C, supernatants were collected and stored at −40°C until use, and cells were resuspended in 1 mL of MEM. The total cell numbers were determined by enumerating them with an Improved Neubauer haemocytometer after being stained with 0.1% trypan blue. For the differential cell counting, cytocentrifuge preparations were made, air dried, and stained with modified Wright's stain. Differential cell counts were performed for a minimum of 500 cells. The results were expressed as absolute numbers of each cell type per mouse peritoneum.

For the experiments investigating mast-cell-migration mechanisms, groups of mice were pretreated intravenously (tail vein injection) with monoclonal antibodies against the adhesion molecules L-selectin, CD11a, CD18, and ICAM-1 (all at a dose of 1 mg·kg^−1^) [29–31], respectively, for 30 min before intraperitoneal injection of 5 *μ*g of promutoxin. Control animals received an equivalent dose of the corresponding normal rat or hamster IgG isotype control. At 6 h following injection, the mice were sacrificed and their peritoneal lavages were processed as described above.

To investigate potential mechanisms involved in promutoxin-induced mast-cell accumulation, several anti-inflammatory compounds including cyproheptadine (2 mg·kg^−1^) [17], terfenadine (3 mg·kg^−1^) [32, 33], ginkgolide B (5 mg·kg^−1^) [34], and quinacrine (10 mg·kg^−1^) [35] were coinjected into the peritoneum of mice with promutoxin (5 *μ*g per mouse). Control animals received an injection of drug alone. At 6 h following injection, mice were sacrificed and their peritoneal lavages were processed as described above. 

### 2.4. Statistical Analysis

Data are shown as mean ± SE for the number of experiments indicated, and ANOVA followed by Tukey's tests were used for statistical comparison of the data. In all analyses, *P* < 0.05 was taken as statistically significant.

## 3. Results

### 3.1. Purification and Characterization of Promutoxin

Approximately 25 mg of promutoxin was obtained from 1.5 g* Protobothrops mucrosquamatus *crude venom following the procedures described above. The purity of the PLA_2_ was at least 98% as assessed by SDS-PAGE, HPLC, and mass spectrometry analysis [24].

### 3.2. Induction of Mast-Cell Accumulation by Promutoxin

**Figure 1:**
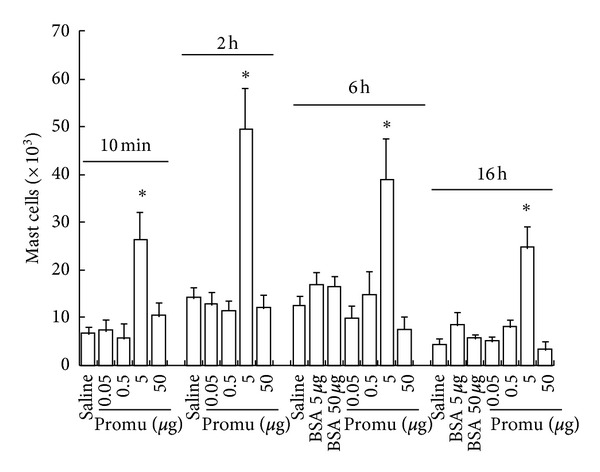
Effect of promutoxin (promu) on mast-cell numbers in mouse peritoneum. Various doses of promu were injected into the peritoneum of mice for 10 min, 2 h, 6 h, or 16 h. Also shown are the responses to BSA and normal saline control. The values shown are mean ± SE for 6 animals in each group. *P < 0.05 compared with the response to the corresponding diluent-only control animals.

As early as 10 min following injection, 5 *μ*g of promutoxin was able to induce significant mast-cell accumulation in the peritoneum of mice. The mast-cell accumulation appeared to last for 16 h (Figure 1). Approximately, up to 6-fold increase in mast-cell numbers was achieved at 16 h following injection (Figure 1).

### 3.3. Effects of Anti-Inflammatory Compounds and Blocking Antibodies on Mast-Cell Accumulation

When coinjected, ginkgolide B, cyproheptadine and terfenadine inhibited 35.9, 71.3, and 92.6% promutoxin-induced mast-cell accumulation in the peritoneum of mice, respectively. However, quinacrine did not significantly alter the extent of promutoxin-induced mast-cell accumulation. At the dose tested, ginkgolide B, cyproheptadine, terfenadine, and quinacrine by themselves failed to induce mast-cell accumulation in the peritoneum of mice (Table 1).

Intravenous injection of monoclonal antibodies against CD18, ICAM-1, and CD11a 30 min prior to intraperitoneal injection of the PLA_2_-blocked promutoxin-induced mast-cell accumulation by 87.2, 76.7, and 53.8%, respectively. Monoclonal antibody against L-selectin failed to diminish promutoxin-induced mast-cell accumulation. Normal rat and hamster IgG-isotype controls tested had little effect on promutoxin-induced mast-cell accumulation (Table 2).

## 4. Discussion

It is found for the first time that promutoxin, a novel member of a minor subgroup (R49 PLA_2_) of snake-venom group II PLA_2_s, can induce mast-cell accumulation. The observation supports our previous finding that N49 PLA_2_, another minor subgroup of snake-venom group II PLA_2_s [23] can induce mast-cell accumulation. Obviously, promutoxin does not induce mast-cell accumulation in a concentration-dependent manner [24]. We previously found that promutoxin could activate mast-cells. The reduction of mast-cell number induced by promutoxin in higher doses may be due to the activation of accumulated mast-cells by promutoxin. 

Ginkgolide B, a PAF antagonist, inhibited more than 35.9% promutoxin-induced mast-cell migration, indicating that PAF is likely involved in mast-cell migration by promutoxin. This result appears to agree with a previous work which found that PAF may contribute to mast-cell migration induced by TM-N49 [23]. Inhibition of 71.3 and 92.6% promutoxin-induced mast-cell accumulation by cyproheptadine, a histamine/5-HT antagonist, and terfenadine a selective histamine-H_1_-receptor antagonist, implies that histamine is likely to be involved in the above event through its H_1_ receptor. Indeed, we have found previously that promutoxin can activate mast cells to release histamine [24] and anticipate herein that released histamine then elicits mast-cell migration. The fact that terfenadine inhibited promutoxin-provoked mast-cell recruitment to a greater extent than cyproheptadine further implies the selectivity of histamine receptor involved. It was found that snake-venom promutoxin could induce mast-cell accumulation even at 10 min following injection, but the number of lymphocyte, macrophage, eosinophil, and neutrophil migration was not altered at 10 min following injection of promutoxin [25]. It seemed the accumulation of neutrophils, lymphocyte, macrophage, and eosinophil induced by promutoxin could be a secondary event, in which accumulated mast cells are activated by promutoxin and release their chemoattractant factors such as serine proteinases [36, 37], histamine, and PAF to recruit inflammatory cells. 

ICAM-1 appears to be a key adhesion molecule of promutoxin-induced mast-cell accumulation as an antibody against ICAM-1 blocked more than 76.7% of the influence of promutoxin on the cell migration. ICAM-1 involved in lymphocyte [38], macrophage [39], eosinophil [40, 41] and mast-cell accumulation [23] has been reported previously, which may support our current observations. An antibody against CD11a (LFA-1) blocked more than 53.8% promutoxin-induced mast-cell accumulation, indicating that CD11a plays an important role in the migration of mast cells, which is consistent with previous reports that TM-N49-induced mast-cell accumulation is mediated by CD18, CD11a, and ICAM-1 [23]. As expected, antibody against CD18 blocked more than 87.2% promutoxin-induced mast-cell accumulation in the present study. Though L-selectin is involved in the neutrophil and eosinophil accumulation provoked by promutoxin, it seemed that L-selectin is not involved in the promutoxin-induced mast-cell accumulation. 

Promutoxin, as a novel member of minor subgroup of PLA_2_, is an enzymatically inactive enzyme. It induced mast-cell accumulation via a PAF, and histamine H_1_ receptor-dependent mechanism, and through a CD11a/CD18-and ICAM-1-associated adhesion pathway. Accumulated mast cells may contribute to snake-venom wound.

## Figures and Tables

**Table 1:** The influence of anti-inflammatory compounds on promutoxin- (5 *μ*g) induced mast-cell accumulation in mouse peritoneum.

Compound injected	Number of mast cells (×10^3^)
Saline	14.6 (6.1–23)
Promutoxin	44.6 (21.4–60.8)
Ginkgolide B 5 mg*·*kg^−1^	26.2 (9.1–41)
Ginkgolide B 5 mg·kg^−1^ + promutoxin	28.6 (13–50.2)*
Cyproheptadine 2 mg*·*kg^−1^	6.2 (1.5–17.2)
Cyproheptadine 2 mg·kg^−1^ + promutoxin	12.8 (4.2–22.2)*
Terfenadine 2 mg·kg^−1^	11.0 (5.8–20)
Terfenadine 2 mg·kg^−1^ + promutoxin	3.3 (1.6–6.5)*
Quinacrine 10 mg·kg^−1^	11.5 (5.9–18.1)
Quinacrine 10 mg·kg^−1^ + promutoxin	36.8 (25.3–49.3)

The values shown are medians (range) for six separate experiments. Compounds were injected into the mouse peritoneum for 6 h before peritoneal lavage fluid was collected. ^∗^ *P* < 0.05 compared with the response to promutoxin alone.

**Table 2:** The influence of blocking antibodies (Ab) against cell-adhesion molecules on promutoxin- (5.0 *μ*g) induced mast-cell accumulation in mouse peritoneum.

Compound injected	Number of mast cells (×10^3^)
Saline	14.6 (6.1–23)
Promutoxin	44.6 (21.4–60.8)
L-selectin Ab + promutoxin	38.0 (18.6–69.0)
LFA-1 Ab + promutoxin	20.6 (9.1–40)*
CD18 Ab + promutoxin	5.7 (3.1–11.7)*
ICAM-1 Ab + promutoxin	10.4 (2.9–14.5)*
Hamster IgG1 + promutoxin	39.8 (17.0–63.1)
Rat IgG2a + promutoxin	43.3 (19.3–58.0)

The values shown are medians (range) for six separate experiments. Monoclonal antibodies (all at a dose of 1 mg·kg^−1^) against the adhesion molecules L-selectin, LFA-1, CD18 and ICAM-1 were intravenously injected, respectively, for 30 min before intraperitoneal injection of 5 *μ*g promutoxin for 6 h. ^∗^ *P* < 0.05 compared with the response to promutoxin alone.
